# A novel easy-to-desorb eluant contributes to address environmental contamination of African swine fever virus

**DOI:** 10.1186/s13568-024-01697-1

**Published:** 2024-05-10

**Authors:** Li Zhang, Pengfei Zhao, Yingjun Xia, Yanli Hu, Chaofei Wang, Rui Fang, Junlong Zhao

**Affiliations:** 1 State Key Laboratory of Agricultural Microbiology, College of Veterinary Medicine, Huazhong Agricultural University, Wuhan, Hubei 430070 China; 2 Wuhan keweichuang biology science and technology co., ltd., Wuhan, Hubei 430076 China; 3 Key Laboratory of Preventive Veterinary Medicine in Hubei Province, Wuhan, Hubei 430070 China; 4 Key Laboratory of Animal Epidemical Disease and Infectious Zoonoses, Ministry of Agriculture, Huazhong Agricultural University, Wuhan, Hubei 430070 China

**Keywords:** ASFV Eluant, Environmental samples, ASFV detection, Environmental surveillance

## Abstract

African swine fever virus (ASFV) is a highly pathogenic and rapidly disseminated virus with strong viability in the environment, suggesting the importance of environmental detection for prevention and control in all the pig industry. However, the detection results of environmental swabs cannot always reflect the accurate status of viral pollution, leading to persistent ASFV environmental contamination. In this study, we developed an ASFV eluant with higher environmental ASFV detection efficiency relative to 0.85% saline solution, which obtains the patent certificate issued by the China Intellectual Property Office (patent number:202010976050.9). qPCR analysis showed that in the environmental swab samples, the number of viral copies was 100 times higher for the ASFV eluant treatment than the traditional eluant treatment (0.85% saline solution). And besides, the high sensitivity of the ASFV eluant had be verified in a slaughterhouse environmental sampling detection. In soil samples, the ASFV eluent showed the same extraction effect as the TIANamp Soil DNA Kit, in contrast to no extraction effect for 0.85% saline solution. Simultaneously, this eluent could protect ASFV from degradation and allow the transportation of samples at ambient temperature without refrigeration. In clinical practice, we monitored the environmental contamination condition of the ASFV in a large-scale pig farm. The results shown that the ASFV load decreased after every disinfection in environment. This study provides an effective solution for surveilling the potential threat of ASFV in environment.

## Key points

1. The ASFV eluant can significantly improve the positive detection rate in environmental samples in clinical practice.
2. The ASFV eluant is economical and convenient compared to commercial kit..
3. The ASFV eluant can provide a caution for the environmental safety for China’s pig industries, slaughter houses, and transportations and facilitate ASFV eradication.

## Introduction

African swine fever virus (ASFV), an enveloped virus with a 180 kb double-stranded DNA, belongs to the sole member of the *Asfarviridae* family (Galindo and Alonso 2017; Bai et al. 2019; Wang et al. 2019), and this virus possesses high infectivity, strong adaptability and devastating force, causing massive damage to the pig industry and economic losses in many countries around the world (Zhao et al. 2019). Currently, there is no vaccine or effective treatment for ASFV, and it is mainly controlled by strict sanitary measures and slaughter (Revilla et al. 2018). Due to anthropogenic factors, the ASFV transboundary and transcontinental diffusional capacity is considerable (de La Rocque et al. 2011), leading to a global ASFV pandemic.

After 2010, the emergence of lower virulent natural mutants brings greater difficulty to early ASFV diagnosis and creates new challenges for its control (Sun et al. 2021), requiring a thorough cleaning and disinfection in the infected sheds and premises before restocking from a known source of healthy farms 4 months later (Patil et al. 2020). The survival of a pathogen in the environment is a key factor to biosafety, and ASFV can survive in most environments, such as water, soil, fodder, meat product, pigpen cement floor, and pigpen walls, thus increasing the difficulty of cleaning and disinfection (Bellini et al. 2016). Periods of ASFV survival were estimated in faeces and urine contaminated with ASFV, as up to eight and 15 days at 4 °C, respectively and five days at 21 °C (Davies et al. 2017); meanwhile, it can also be transmitted through water and survive there at different temperatures and light conditions (Karalyan et al. 2019); ASFV does not show significant changes in viral titers within 25 h at room temperature (Turner and Williams 1999). The main ASFV transmission routes were reported as direct contact with infectious and susceptible domestic pigs, as well as indirect contact with contaminated pork, people, vehicles, and fomites (Penrith and Vosloo 2009; Nielsen et al. 2021). According to statistics from the Ministry of Agriculture and Rural Affairs of China, from August 2018 when the African swine fever outbreak was reported until November 2018, the rapid spread of African swine fever was attributed to infected pigs or pork products accounting for 19%, swill feeding accounting for 34%, and personnel and vehicle movement accounting for 46% (Dixon et al. 2019); the main factors influencing the progressive spread of ASF in the Russian Federation are illegal movement of pork products, untreated swill feeding, free-ranging pig production practices and personnel movement (Gogin et al. 2013); Mur et al. proposed that ASFV can readily spread through waste from returning trucks, waste generated by international vessels, and garbage from international flights and they have developed a risk analysis model to assess the likelihood of such transmission (Mur et al. 2012). Briefly, the contaminated environment plays a significant role in continuous zonal transmission, indicating that laboratory detection has become an essential means to ensure accurate environmental disinfection during production resumption and ASFV decontamination. Despite its tenacious survival in most environments, ASFV can be hardly detected by qPCR in most environment samples, causing the omission of positive samples and posing a threat to the pig industry. The low detection rate of environmental samples in Chinese grassroots pig farms can be primarily attributed to factors such as inadequate and careless environmental sampling, lengthy sample transportation time, and insufficient virus enrichment in traditional sampling fluids. Therefore, we collected a mass of information about how to gather and elute virus and increase the detection rate of pathogens. For example, some researchers used the solution of acidic AlCl_3_ to elute the virus and obtained 90% rotavirus, 20% adenovirus and 100% hepatitis A virus (Schlindwein et al. 2009). Additionally, the elution efficiency was shown to be significantly enhanced by increasing the alkalinity of glycine for the virus (Hurst et al. 1991). Generally, the virus eluent consists of distilled water, gelatin, fetal bovine serum, sodium doddery sulfate (SDS) and glycine (Albert et al., 1991; Grabow et al., 1991; Monpoeho et al. 2001). Trehalose is shown as a better choice for prevention of ASFV degradation, due to its role as a stabilizer for liquid vaccines of classical swine fever (CFS) (Williamson et al. 2003), as well as its significant stabilizing effects on viral protein tertiary structure or membrane integrity (Kissmann et al. 2011).

The objectives of this study is to develop a reagent capable of collecting and eluting ASFV for production resumption and ASF decontamination in the environment. Several reagents with a potneial affinity for ASFV were screened and optimized at different concentrations and used to elute tissue, soil and blood swabs to verify their eltution effects.

## Materials and methods

### Detection and plasmid construction of ASFV

The qPCR method based on ASFV p72 gene was established for accurate testing of environmental swabs, and the primers used are shown in Table 1. The automatic nucleic acid extractor used for DNA extraction was purchased from Luoyang Ascend Biotechnology Co., Ltd (Henan, China). The TIANamp Soil DNA Kit was purchased from Tiangen Biotech CO., LTD (Beijing, China). The qPCR was performed under the conditions of 1 cycle at 25 ℃ for 10 min for uracil-N-glycosylase activation, 1 cycle at 95 ℃ for 20 s for predegeneration, followed by 45 cycles at 95 ℃ for 1 s for degeneration and 58 ℃ for annealing/extension.

**Table 1:** Primers used for qPCR in the study

Primer	Sequence (5′-3′)
P72-F	GATACCACAAGATCAGCCGT
P72-R	TCGATAAGATTGATACCATGAGCAG
P72-probe	CCACGGGAGGAATACCAACCCAGTG

The reference sequence of p72 gene, the detected samples in our lab, was used to design the primers, and then the target sequence (the sequence of 103,682 to 103,931) was cloned into the pEASY-blunt to generate a pEASY-blunt-p72 recombinant plasmid. The precise viral load in the positive samples was determined using the solutions of the pEASY-blunt-p72 plasmid diluted in the range of 10^1^ to 10^10^. The qPCR runs of the experimental samples involved three replicates.

### Different types of sample processing methods

For qPCR detection of environmental samples, 500 µL ASFV eluant and sterile cotton swabs were prepared, followed by using the cotton swabs dipped with the ASFV eluant to wipe the sampling area repeatedly, squeezing the cotton swabs along the tube wall and centrifugation for 3 min at 14,000 g to collect the supernatant. Finally, the DNA was extracted from the supernatant for qPCR analysis.

For qPCR detection of soil samples, 2 steel balls and 500 µL ASFV eluant are mixed into the tissue in a tube, followed by oscillating the tube for 10 min and centrifugation for 3 min at 14,000 g to to collect the supernatant for DNA extraction and qPCR detection.

For qPCR detection of blood swab samples, the blood swabs were dipped in 500 µL ASFV eluant for 10 min, followed by squeezing the swabs along the tube wall to collect solution for DNA extraction and qPCR detection. The ASFV eluant was used to desorb ASFV from swabs and protect ASFV from degradation.

### Ingredients of the ASFV eluant

The ASFV eluant, a mixed solution of glycine, SDS, pure water and D-trehalose dehydrate, can desorb ASFV from environmental samples. Similarly, these ingredients embodied in the patent certificate.

Firstly, reagents that may aid in the collection and elution of ASFV from environmental samples were screened: glycine-sodium hydroxide solution, SDS, BSA, and FBS.

Then several reagents with virus enrichment effects were combined in order to discover synergistic effects and further enhance the ability to enrich and elute ASFV from the environment and environmental samples. Due to difficulties in dissolving FBS with other reagents, FBS was excluded from the collection solution formulation. The other reagents were combined into three different combinations: glycine-sodium hydroxide solution with 10% SDS, glycine-sodium hydroxide solution with 10% BSA, and glycine-sodium hydroxide solution with 10% SDS and 10% BSA. These 3 combinations were used to prepare the collection solution for collecting simulated environmental samples. After nucleic acid extraction using an automated nucleic acid extractor, qPCR was performed for detection.

A preliminary environmental sample collection solution was prepared based on a 7.5 mg/ml glycine-sodium hydroxide solution. It was combined with a protein-lipid soluble substance solvent of 4 mg/ml SDS and a bioprotectant of 5 mg/ml trehalose (trehalose acts to protect viral nucleic acid from degradation and does not affect the elution efficiency of the established collection solution, data not shown). To optimize the proportions of the components, 6 different concentrations of collection solutions were prepared as follows:

1. 1.875 mg/mL glycine-sodium hydroxide solution, 1 mg/mL SDS, 1.25 mg/mL trehalose.
2. 3.75 mg/mL glycine-sodium hydroxide solution, 2 mg/mL SDS, 2.5 mg/mL trehalose.
3. 7.5 mg/mL glycine-sodium hydroxide solution, 4 mg/mL SDS, 5 mg/mL trehalose.
4. 15 mg/mL glycine-sodium hydroxide solution, 8 mg/mL SDS, 10 mg/mL trehalose.
5. 22.5 mg/mL glycine-sodium hydroxide solution, 12 mg/mL SDS, 15 mg/mL trehalose.
6. 30 mg/mL glycine-sodium hydroxide solution, 16 mg/mL SDS, 20 mg/mL trehalose.

(The concentrations of the reagents in the preliminary environmental sample collection solution were diluted 1 and 2 times, and 2, 3, 4 times were added.)

#### Desorption effect of virus from environmental samples

Briefly, a cement floor was partitioned into different zones with an area of 1 × 1 cm^2^, followed by dropping the positive virus blood samples (100 µL each with 5 to 55-fold dilution) as simulated environmental samples. 24 h later, the positive environmental areas were eluted separately by swabs dipped with the eluant and 0.85% saline solution to collect the environmental samples, followed by qPCR analysis of the virus concentration differences between the ASFV eluant and 0.85% saline solution. The qPCR analysis was repeated 3 times for each experimental sample, and the average value was used for data analysis. Before dropping the positive virus blood samples, the cement floor was detected negative, and each zone was used only once in the test of environmental samples.

### Desorption of virus from soil samples

Soil samples were prepared by dropping 100 µL positive blood samples into 0.2 g soil and incubation for 24 h, followed by treating the soil samples with the ASFV eluant and the TIANamp Soil DNA Kit *via* mixing 500 µL ASFV eluant and 2 steel balls with the soil in a tube. Then, the tube was oscillated for 10 min and centrifuged for 3 min at 14,000 g to obtain the supernatant for DNA extraction and qPCR detection. The qPCR was performed for each experimental sample with 5 replicates, and the average value was used for data analysis.

### Desorption and protection of virus in blood swabs

In the blood swab experiment, cotton swabs were dipped in a positive blood sample and put in a tube, followed by treating the simulated blood swabs at 37 ℃ for 24, 48 and 72 h just like the case of sending samples to a test station from a pig farm. The experimental samples were divided into 2 groups by treating the simulated blood swabs separately with the eluant and saline solution, where the blood swabs were squeezed along the wall of each tube to collect the solution for DNA extraction and qPCR detection to verify the protective efficacy of the ASFV eluant.

#### Comparison of the ASFV eluant and saline solution in detection of clinical samples

A comprehensive collection of 102 environmental samples was conducted at a slaughterhouse in Hubei, China, utilizing swabs immersed in the eluant and 0.85% saline solution. These samples encompassed various elements such as transport vehicles, production equipment, and human bodies, enabling a comparative analysis of the test outcomes between the 2 treatment methods.

#### Clinical application of the ASFV eluant

Environmental samples were collected by swabs dipped in the ASFV eluant to test the effect of the eluant in clinical practice. A total of 175 environmental swabs were collected from a large-scale pig farm in Hubei, China and detected by the ASFV eluant. There were 17 categories of environment samples collected by the ASFV eluant after the pigs developed symptoms of ASFV and samples of these categories were collected again after 3 thorough disinfection. Furthermore, the trestles of farrowing bed, the most contaminated area, were tracked and detected to reveal the disinfection effect.

## Results

### Detection and plasmid construction of ASFV

**Fig. 1:**
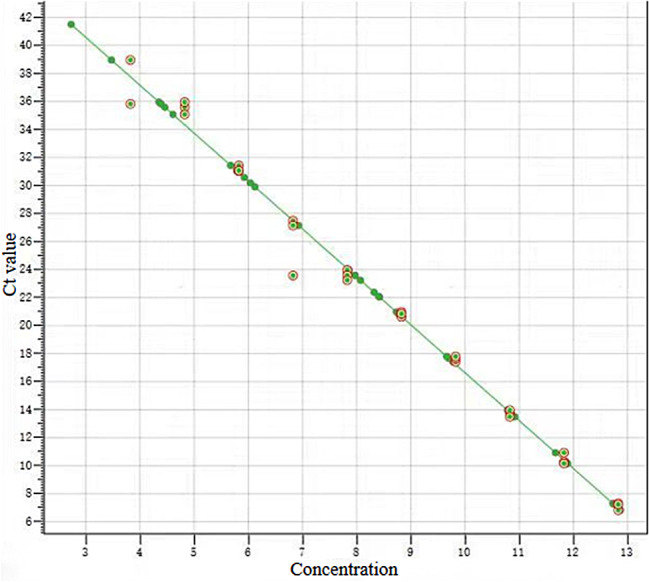
The standard curve of the pEASY-blunt-p72 recombinant plasmid (6.69 × 10^13^) copies/ml, with the concentrations of positive samples calculated from the Ct values

According to the qPCR results, a standard curve was made based on pEASY-blunt-p72 recombinant plasmid (6.69 × 10^13^) copies/mL, and the concentration of positive samples was calculated using the Ct values (Fig. 1). The standard curve is y=-3.42x + 50.83.

### Ingredients of the ASFV eluant

**Fig. 2:**
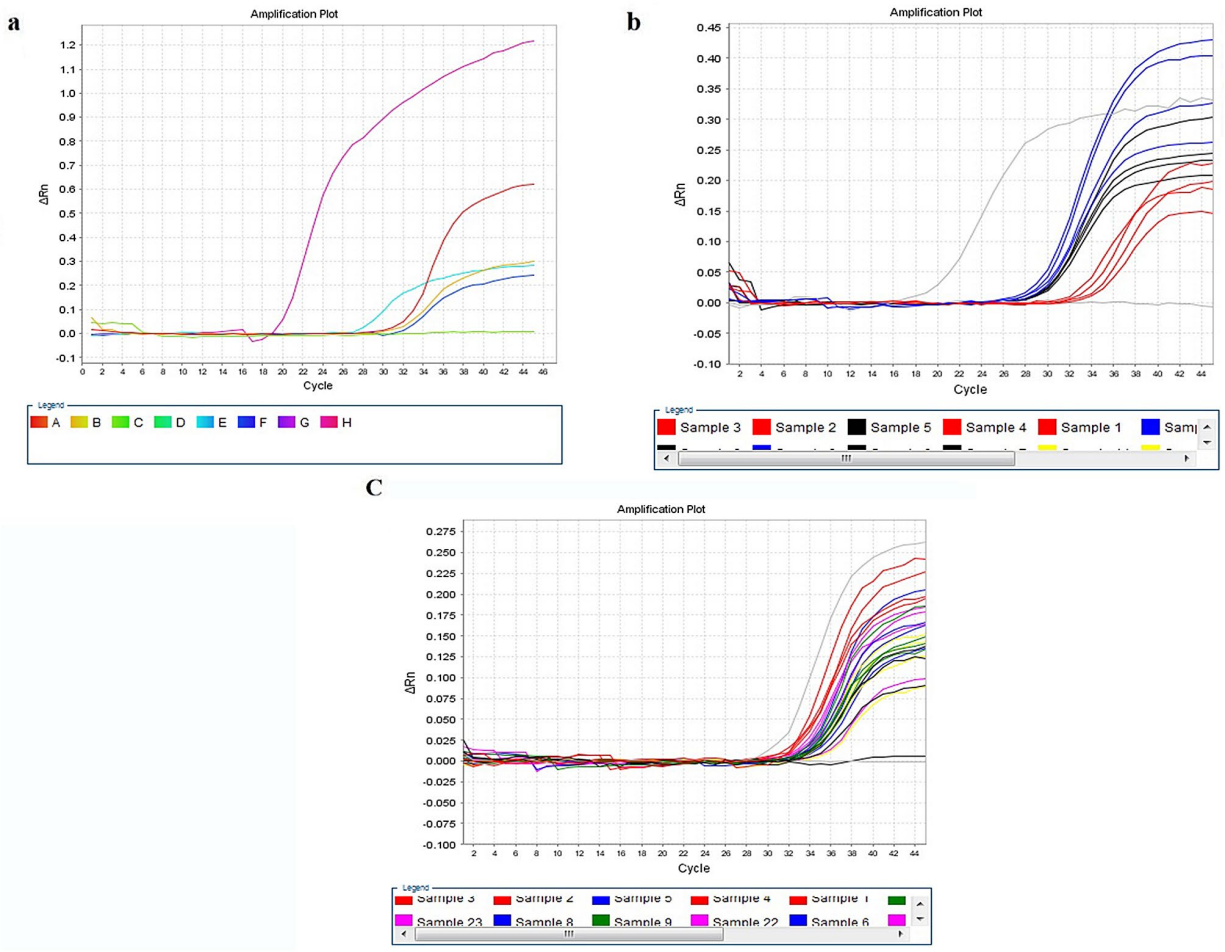
Ingredient screening and formulation optimization of the ASFV eluant. (**a**) Preliminary screening of collection solution ingredients, Red, 10% sodium dodecyl sulfate, Light Blue, glycine-NaOH solution, Yellow, 10% fetal bovine serum, Dark Blue, 10% bovine serum albumin, Green, 0.85% saline solution. (**b**) Compatibility testing of collection solution ingredients, Blue, glycine-NaOH solution, sodium dodecyl sulfate, Black, glycine-NaOH solution, sodium dodecyl sulfate, bovine serum albumin, Red, glycine-NaOH solution, bovine serum albumin. (**c**) Yellow, 1.875 mg/mL glycine-NaOH solution, 1 mg/mL SDS, 1.25 mg/mL trehalose, Black, 3.75 mg/mL glycine-NaOH solution, 2 mg/mL SDS, 2.5 mg/mL trehalose, Purple, 7.5 mg/mL glycine-NaOH solution, 4 mg/mL SDS, 5 mg/mL trehalose, Green, 15 mg/mL glycine-NaOH solution, 8 mg/mL SDS, 10 mg/mL trehalose, Red, 22.5 mg/mL glycine-NaOH solution, 12 mg/mL SDS, 15 mg/mL trehalose, Blue, 30 mg/mL glycine-NaOH solution, 16 mg/mL SDS, 20 mg/mL trehalose

The preliminary screening identified four reagents that exhibited a certain collection and elution ability for African swine fever virus in the environment (Fig. 2a), with the glycine-sodium hydroxide solution performing the best and 10% BSA showing the least significant effect. To investigate whether there is a synergistic effect, we combined these reagents and dissolved them in pure water for analysis as the environmental sample collection solution. The combination of glycine-sodium hydroxide solution and SDS achieved the best results, while the other two combinations containing BSA did not show better performance than those without BSA (Fig. 2b). Therefore, we selected glycine-sodium hydroxide solution and SDS as the components of the environmental sample collection solution. To ensure the stability and prevent degradation of the African swine fever virus nucleic acid during the long-distance transportation of samples before laboratory testing, we incorporated trehalose as a protective agent. The addition of trehalose did not affect the original collection and elution efficiency of the environmental sample collection solution.

Finally, based on the preliminary formulation of 7.5 mg/mL glycine-sodium hydroxide solution, 4 mg/mL SDS, and 5 mg/mL trehalose, we optimized the composition ratio of the developed environmental sample collection solution by diluting the components 1 and 2 times and adding 2, 3, and 4 times. From Figs. 5-1c, it can be observed that the composition ratio of 22.5 mg/mL glycine-sodium hydroxide solution, 12 mg/mL SDS, and 15 mg/mL trehalose enriched and eluted more viruses, indicating it as the optimal composition ratio.

### Detetion of environmental swabs

**Fig. 3:**
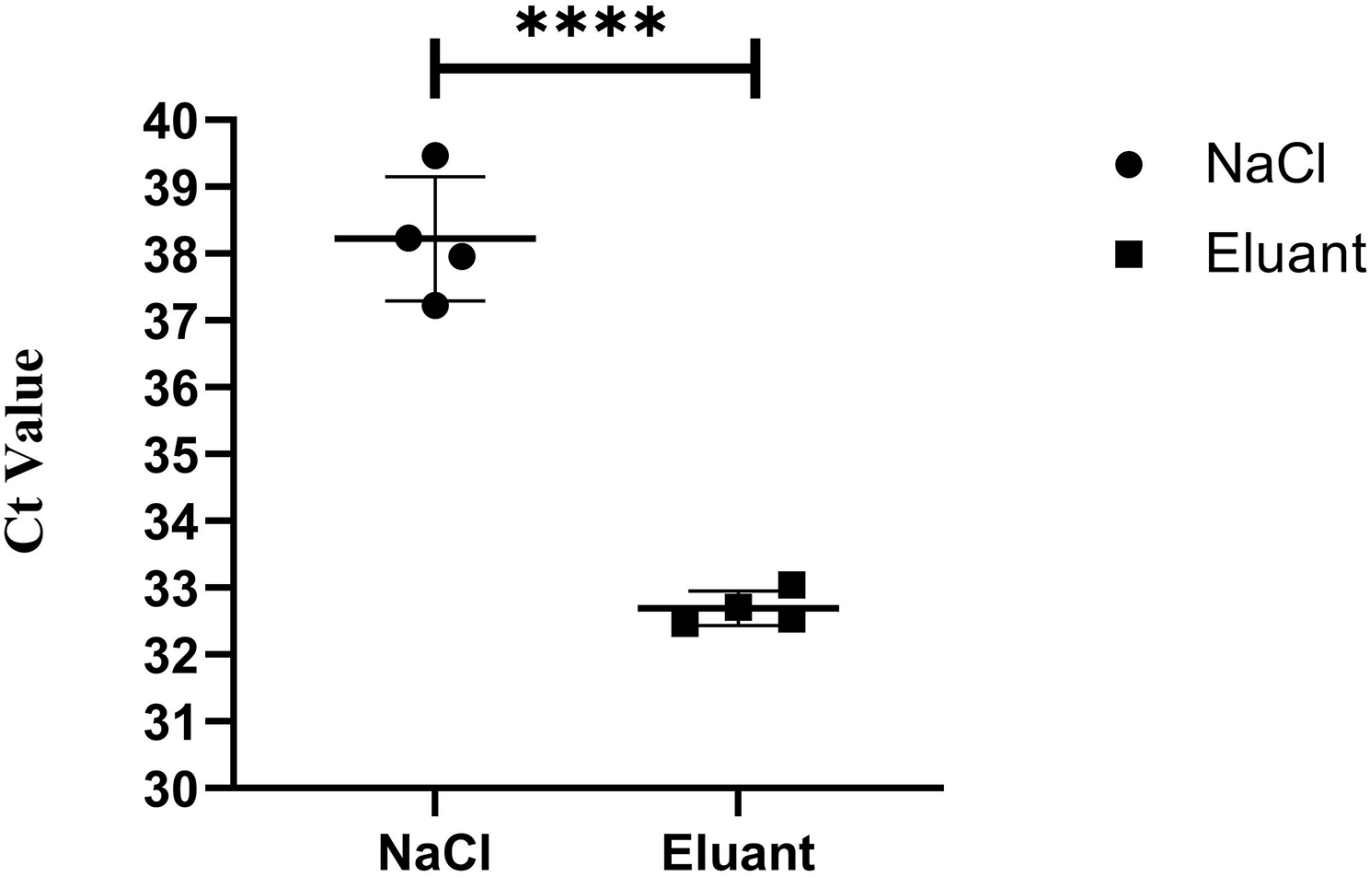
The ASFV eluant demonstrates superior performance in the collection and elution of ASFV compared to 0.85% saline solution

The qPCR results indicated that the Ct value of swabs was only 38.05 (3.7 × 10^4^ copies) for the positive samples with 5^− 2^-fold dilution in group 2 (collected and eluted by 0.85% saline solution), in contrtast to the Ct value of 31.63 (2.7 × 10^6^ copies/µL) for the samples collected and desorbed by the ASFV eluant (Table 2). Furthermore, the ASFV eluant was compared with 0.85% saline solution in the detection limit of saline solution (*n* = 4). The results indicated the ASFV eluant had better ASFV elution effect, with a higher viral content and a decrease of ~ 6 Ct values in group 1 (ASFV eluant) relative to group 2 (0.85% saline solution) (Fig. 3).

**Table 2:** Elution effects of environmental swabs

Dilution ratio^(a)^	Collection and elution by the ASFV eluant (Ct)	Collection and elution by 0.85% saline slution (Ct)	*p*
5^− 1^ (28.41 ± 0.47)	30.84 (± 0.88)	34.40 (± 1.34)	*p* < 0.01
5^− 2^ (29.77 ± 0.71)	31.63 (± 0.31)	38.70 (± 1.61)	*p* < 0.001
5^− 3^ (31.97 ± 0.24)	33.99 (± 0.27)	38.63 (± 1.09)	*p* < 0.003
5^− 4^ (35.23 ± 0.46)	36.85 (± 0.31)	/	/
5^− 5^ (37.46 ± 1.32)	/	/	/

Results are shown as mean ± standard deviation (SD) (*n* = 3) and the difference between two groups was determined at *p <* 0.05 by the t-test; (a) Ct values of blood before simulation

### Detetion of virus in soil samples

**Fig. 4:**
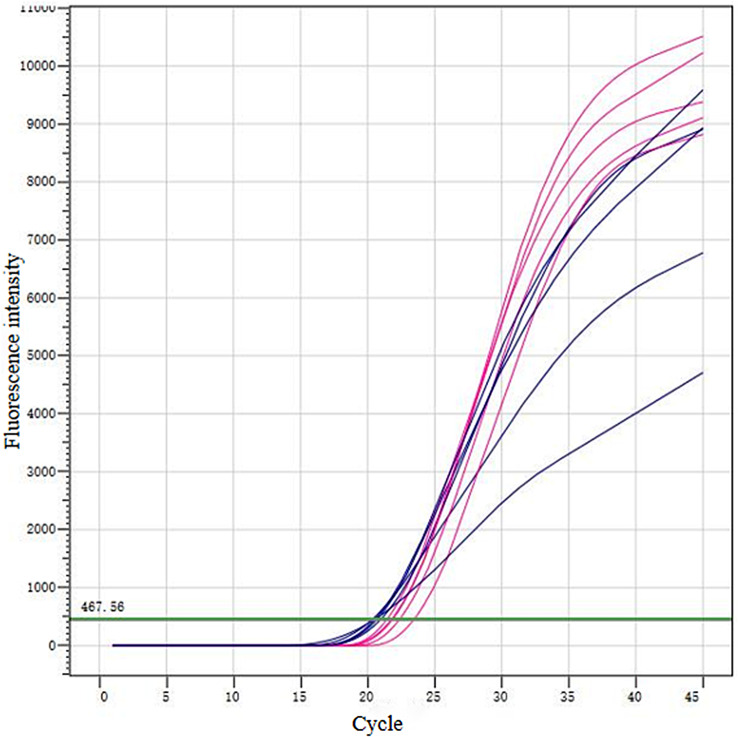
The comparison of samples desorption effect for the ASFV eluant and the TIANamp Soil DNA Kit, with red for TIANamp Soil DNA Kit and blue for the ASFV eluant

The results indicated that the eluant had a similar or even a better performance than the kit. In Fig. 4, the kit was seen to substantially decontaminate the impurities of the samples and form a more standard amplification curve, while the eluant only involves the steps of oscillation and centrifugation in cleaning the impurity, much simpler than the kit in the cleaning process (Fig. 4).

### Detection and protection of virus in blood swabs

In the test of blood swabs, the viral copies stayed at the same level in the ASFV eluant group throughout the test, in contrast to an obvious decrease (*p* < 0.05) in the 0.85% saline solution group between 36 and 72 h (Table 3).

**Table 3:** Elution effects of blood swabs

Time (h)	Blood swabs mixed with the ASFV Eluant (Ct)	Blood swabs mixed with 0.85% Saline slution (Ct)	p values
0	37.07 (± 1.04)	36.44 (± 0.33)	*p* < 0.5
24	35.91 (± 0.58)	37.96 (± 0.91)	*p* < 0.05
36	37.07 (± 0.50)	/	*/*
72	35.37 (± 0.61)	38.25 (± 1.76)	*p* < 0.05

Results are shown as mean ± standard deviation (SD) (*n* = 3), and the difference between two groups was determined at *p <* 0.05 by the t-test

### Comparison of saline solution and ASFV eluant in detection of clinical samples

**Fig. 5:**
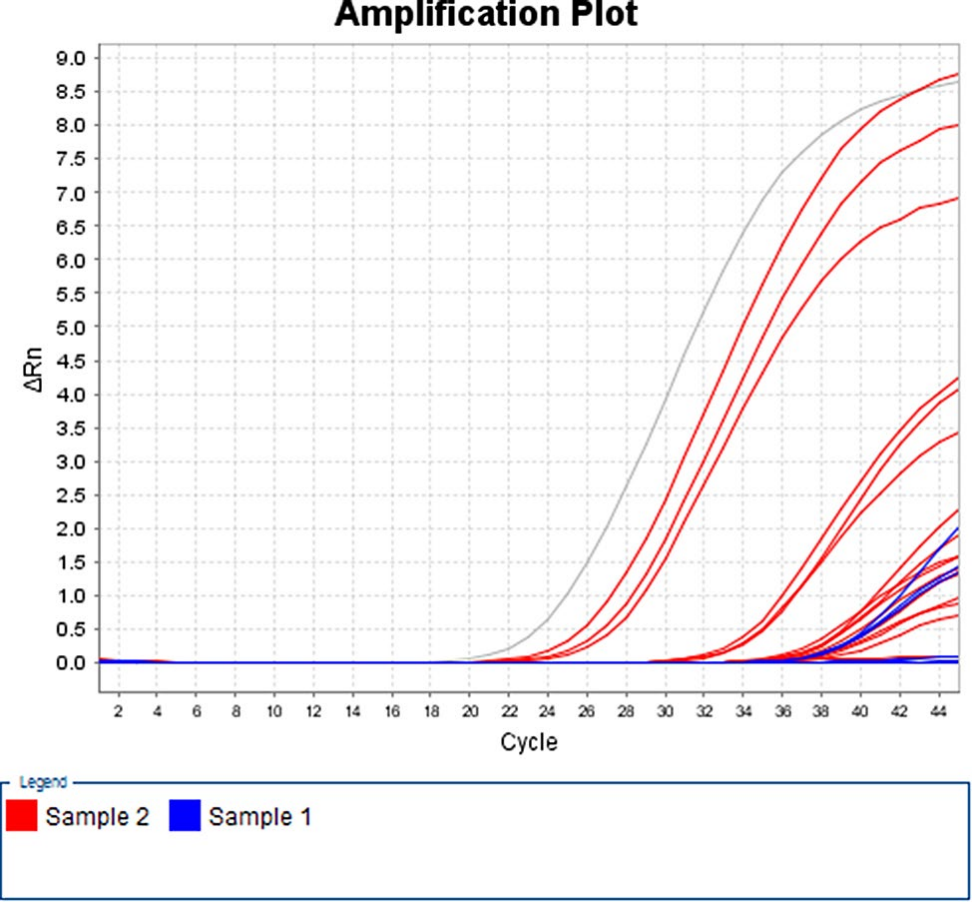
The comparison of effect of samples collection for the ASFV eluant and 0.85% saline solution in clinical practice, with red for the ASFV eluant, blue for 0.85% saline solution and gray for positive control

Three of the 51 clinical samples treated with 0.85% saline solution were detected positive, in contrast to 17 samples detected positive in the same number of samples treated by the ASFV eluant (Fig. 5). Additionally, qPCR results showed that the ASFV eluant has significant advantages over 0.85% saline solution in samples collection of transport vehicle and human body (Table 4).

**Table 4:** Comparison of elution effects between 0.85% saline solution and the ASFV eluent during a sampling and detecting experiment conducted at slaughterhouse

Sampling reagent	Transport vehicle	Human body	Production equipment	Pig body	total
0.85% saline solution (positive rate)	1/6	1/6	1/30	0/9	3/51
the ASFV eluant (positive rate)	6/6	4/6	6/30	1/9	17/51

### Detection of clinical samples

**Fig. 6:**
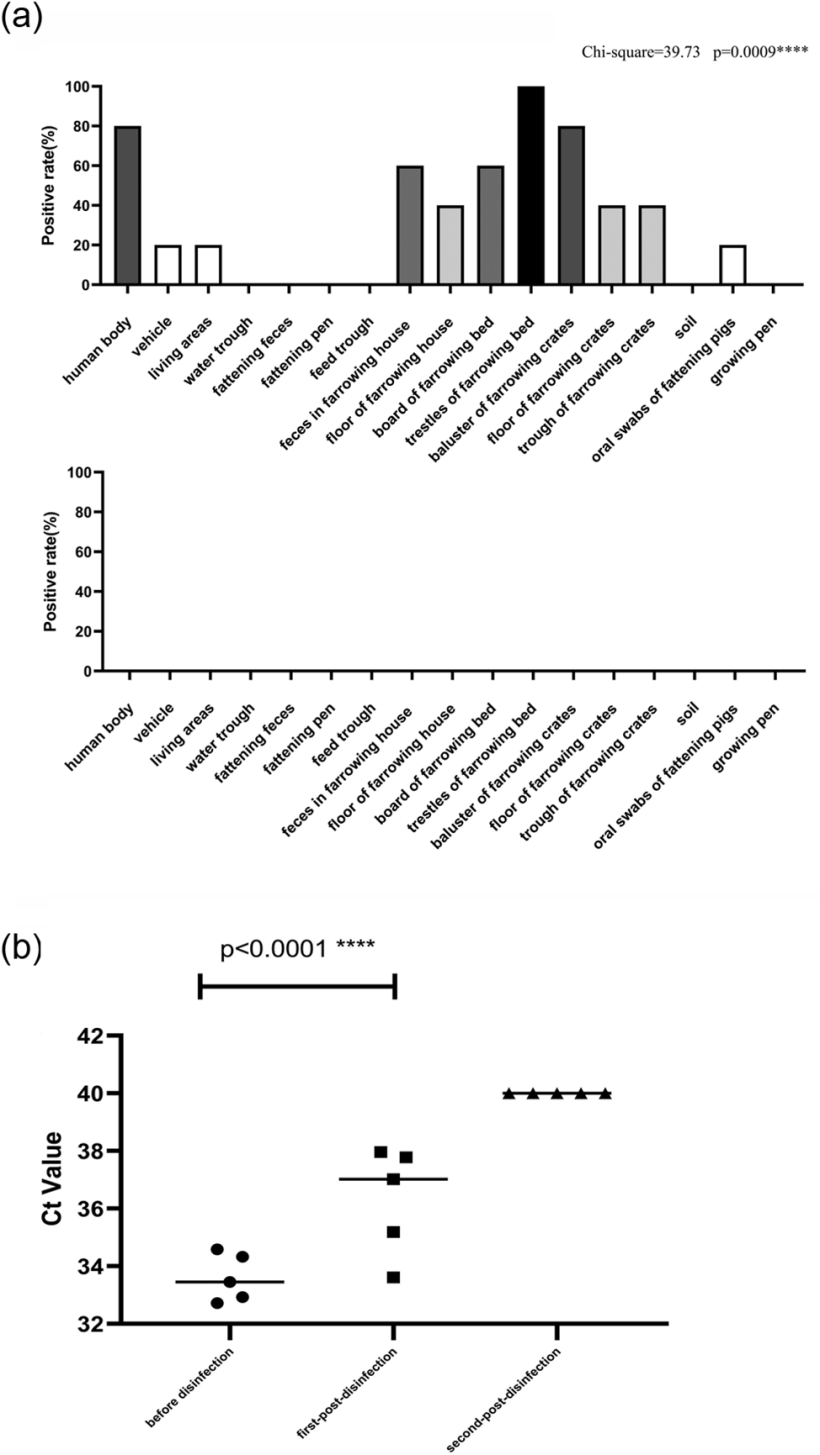
(**a**) The detection results of environmental samples from the pig farm before and after disinfection. (**b**) Evaluation of disinfection effectiveness in the most contaminated areas

In practical application, the qPCR results showed that 28 samples were detected positive in a total of 85 samples after the pigs developed symptoms of ASF. The different shades of Histograms represent the severity of contamination. After thorough disinfection, all of the same categories samples were detected negative (Fig. 6a). The tracking detection reveal that the repeated disinfection is required to achieve virus clearance (Fig. 6b). The ASFV eluant can effectively and accurately monitor the contamination of ASFV in environment.

## Discussion

In this study, the developed ASFV eluant was shown to be more effective in gathering and desorbing the ASFV virus from samples when mixed with other reagents at a varying concentration and proportion. Specifically, trehalose is a non-specific bioactivator and was used as a stabilizer for classical swine fever virus (CSFV) liquid vaccines (Pachauri et al. 2020). The trehalose and SDS have a positive effect on ASFV collection and elution, in contrast to a negative impact of BSA on the qPCR results when mixed in the eluant. Further, the PCR curve can possibly be further improved by developing the ASFV eluant containing humic acid inhibitors (for handling soil samples) or virus inactivators (for reducing environment contamination). Accidentally, we have discovered that the ASFV eluant exhibits outstanding capability in enriching the *Lowsonia intracellularis* in fecal samples, in addition to its effectiveness in African swine fever virus collection and elution (date no shown). As a result, the ASFV eluant holds significant potential for further development.

At present, due to unavailability of effective vaccines, many laboratory detection methods and rapid methods have been developed for ASFV detection (Wang et al. 2017, 2020; Bai et al. 2019). However, ASFV detection is based on samples collection, virus enrichment and nucleic acid extraction. Also, in contrast to experimental studies, the actual process of collecting environmental samples by farm employees may not strictly adhere to sampling protocols after the occurrence of ASF outbreaks in Chinese grassroots pig farms. This, combined with other objective factors, contributes to the problem of a low detection rate of environmental samples. This is reflected in our statistical laboratory analysis of clinical samples from pig farms across various provinces in China (date no shown). Therefore, the advantages of the ASFV eluant lie in its simplicity and ease of operation, strong virus enrichment capability, and protection of viral nucleic acids. Our results indicate that the ASFV eluant had better performance than 0.85% saline solution in gathering, eluting and protecting virus. Additionally, the ASFV eluant can desorb and dissolve ASFV from swabs. For simulative blood swabs, the viral copies remained unchanged at 37 ℃ for 72 h at 200 µL eluant in the experiments, indicating the novel eluant can protect ASFV in the transportation of samples at ambient temperature. Moreover, the *p* values showed significant difference in extracting tissues with a low viral content, while a smaller difference when extracting tissues with a high viral content, demostrating the great advantge of the ASFV eluant in detection of low-viral-content samples, particularly the environmental samples whose virus desorption is difficult during the circulation of low virulent natural mutants in China (Sun et al. 2021). The clinical experimental results proved that the potential threat of ASFV in the environment is undetectable when treating the environmental samples by traditional elution (0.85% saline solution), but can be accurately reflected by the ASFV eluent. Although multiple virus dilutions decreased its effectiveness, the ASFV eluent still shows significant effect in viral collection and extraction in experiment of the ASFV eluant and saline solution in detection of clinical samples. Ultimately, the effect of each disinfection could be clearly and accurately monitored.

In summary, the ASFV eluant exhibits widespread applicability, high accuracy, high sensitivity, simplicity and excellent stability for environmental ASFV detection. The ASFV eluant produces prominent effect in ASFV collection, elution and detection of various samples, such as cement floor swabs, soil samples or blood swabs. Additionally, the ASFV eluant allows the transportion of smples at room temperature, desorption of more virus from samples, and easy ASFV detection in China. Currently, few analytical studies have been performed regarind the risk factors of ASF in environmental safty, including China’s pig industries, slaughter houses, and transportations, and the data from the developed eluant detection could provide a caution for the environmental safety. Therefore, the developed eluant can facilitate ASFV environmental detection and eradication for the pig industry and produce a profound influence on the current ASF epidemic in China.

## Data Availability

All relevant data are within the manuscript and its Additional files.
